# Conceptualizing burnout from the perspective of parents of children with complex care needs

**DOI:** 10.1016/j.pecinn.2024.100325

**Published:** 2024-07-17

**Authors:** Nathalie J.S. Patty, Karen M. van Meeteren, Minke Verdonk, Marjolijn Ketelaar, Carlo Schuengel, Agnes M. Willemen

**Affiliations:** a Faculty of Behavioural and Movement Sciences, Section Clinical and Family Studies, Vrije Universiteit Amsterdam, Van der Boechorststraat 7, 1081 BT Amsterdam, the Netherlands; b Amsterdam Institute of Public Health, Van der Boechorststraat 7, 1081 BT Amsterdam, the Netherlands; c OuderInzicht, Kattegat 1, 1501 AJ Zaandam, the Netherlands; d UMC Utrecht Brain Center, University Medical Center Utrecht, Heidelberglaan 100, 3584 CX Utrecht, the Netherlands; e De Hoogstraat Rehabilitation, Center of Excellence for Rehabilitation Medicine, Rembrandtkade 10, 3583 TM Utrecht, the Netherlands

**Keywords:** Burnout, Parents of children with complex care needs, Parental burnout, Caregiver burnout

## Abstract

**Objective:**

The purpose of this study was to investigate how parents of children with complex care needs conceptualize burnout from the perspective of parents themselves.

**Methods:**

We conducted semi-structured interviews with 38 parents, selected for maximal variation in parental, child, and family characteristics. Inductive thematic analysis was employed.

**Results:**

Burnout was conceptualized as encompassing three themes: having a reoccurring long-term nature, commencing with symptoms of stress progressing into exhaustion, and ending in a survival mode wherein parents worked hard to project an image of everything being well and under control (fighting) while distancing physically and emotionally from others and themselves (fleeing).

**Conclusion:**

Burnout involves specific aspects of caregiving and parenting, such as long-term responsibility for the child, which cannot be relinquished. Furthermore, burnout may also be ‘hidden’: not always showing to the outside world, which requires extra attention and vigilance among parent's informal and formal networks. Awareness of the various interpretations of the term may foster constructive communication.

**Innovation:**

Focusing on parents’ individual experiences has illuminated new aspects of burnout. By purposively sampling a variety of parents of children with complex care needs, a broader understanding of the meaning of the term ‘burnout’ from the perspective of parents was achieved.

## Highlights

• Parents of children with complex care highlight new aspects of burnout.
• Burnout among parents of children with complex care is context specific.
• Burnout among parents may be difficult to detect due to its hidden nature.
• Awareness regarding burnout may foster (in)formal support.

## 1 Introduction

Parents bear the primary responsibility for their children and thus feel the added demands if children have complex care needs (CCN) [1]. Children with CCN have a combination of enduring physical, developmental, behavioral and/or emotional problems and needs. To address these problems and needs, parents tend to go beyond their regular parenting duties to engage in activities such as advocacy, case management, and nursing tasks [2,3]. These tasks may conflict with their personal needs and those of other family members [4]. As a result, parents of children with CCN have reported adverse effects on their wellbeing including elevated stress [5], reduced quality of life [6,7], and heightened levels of depression compared to parents without children with CCN [8,9]. The concept of ‘burnout’ was recently introduced in the scientific literature [10,11] and has been described as a consequence of prolonged exposure to parental stressors and challenges [12]. Within parenting, researchers have linked burnout to grave consequences, such as suicidal thoughts, substance abuse, domestic conflicts, and violence and neglect toward the child [13]. However, it is unclear whether the understanding of parental burnout in the emerging scientific literature aligns with how parents understand this concept. Therefore, this study aims to describe what burnout among parents of children with CCN entails within the context of parenting a child with CCN.

Based on the occupational context [14], burnout within parenting, known as ‘parental burnout’, has received increasing attention in the recent scientific literature [15], including studies within the context of parenting a child with CCN [10,11]. In their review, Patty et al. [11] reported that scientific conceptualizations of burnout in parents of children with CCN included symptoms, onset factors, and pathogenesis. Burnout was sometimes addressed across life domains (such as work, partner relationships, and caregiving) and at other times focused on the specific context of caregiving. Patty et al. [11] proposed that the conceptualization of burnout among parents of children with CCN should be context dependent due to dual parental and caregiving responsibilities. This unique combination distinguishes burnout among parents of children with CCN from burnout within a work-related context, parental context, and a caregiving context. However, a clear understanding of burnout in the context of parenting a child with CCN is lacking, and the perspectives of parents themselves regarding burnout remain largely unexplored.

Several studies have adapted conceptualizations from occupational settings to investigate burnout among parents of children with CCN [e.g., [7], [8], [16], [17], [18], [19], [20]]. Prominent conceptualizations refer to Maslach et al. [21] (the Maslach Burnout Inventory: MBI) and Melamed et al. [22] (the Shirom–Melamed Burnout Questionnaire: SMBQ). The conceptualization by Maslach et al. [21] involves three dimensions: ‘emotional exhaustion’, signifying a depletion of emotional resources; ‘depersonalization’, involving cynical feelings toward others; and ‘lack of personal accomplishment’, where one feels inadequate toward the ability to do the job. Melamed et al. [22], on the other hand, described burnout according to four dimensions: ‘emotional exhaustion and physical fatigue’, signifying feelings of being emotionally and physically drained; ‘listlessness’, denoting lack of energy and lethargy; ‘tension’, identifying restlessness and feelings related to inner tension; and ‘cognitive difficulties’, signifying cognitive weariness.

Acknowledging the potential limitations of applying conceptualizations from an occupational context in a parenting context, Roskam et al. [14] adjusted some of the components identified by Maslach et al. [21] to fit a parenting context, developing the Parental Burnout Inventory (PBI). Their conceptualization encompasses three dimensions of burnout related to the parental role: ‘emotional exhaustion’, which involves intense exhaustion in the parental role; ‘emotional distancing’, where parents disengage emotionally from their child; and ‘lack of personal accomplishment’, indicating a decline in parental effectiveness [14,23]. Recognizing the potential limitations of not incorporating the perspectives of parents, Roskam et al. [24] reconceptualized burnout by interviewing five burnt-out parents. This conceptualization resulted in an adapted inventory (Parental Burnout Assessment: PBA) encompassing four dimensions, two overlapping with the previous conceptualization — (1) ‘emotional exhaustion’ and (2) ‘emotional distancing’ — and two new dimensions — (3) ‘contrast with previous parental self’, measuring the discrepancy parents may notice when they compare themselves with earlier years, and (4) ‘feelings of being fed up’, signifying a decreased pleasure in the role as parents. Research on burnout in the context of children with CCN has often adopted these conceptualizations, even as these pertained to the general population and not necessarily to parents of children with CCN (e.g., PBI: [[25], [26]] PBA: [[27], [28]]).

Studies that used alternative conceptualizations of parental burnout have not always explained their theoretical and empirical foundations [29,30]. These studies conceptualized burnout in terms of helplessness and withdrawal [e.g., [29], [30], [31]]. Acknowledging the several ways that burnout has been conceptualized, Abdoli et al. [32] examined parents' experiences of burnout among parents of children with type 1 diabetes and framed burnout within this context as exhaustion due to persistent stress, feelings of powerlessness, grief over lost normalcy, and active pursuit of coping mechanisms, rooted in the unceasing responsibility of being the primary caregiver for their child’s diabetes care.

In sum, while the concept of burnout has been applied to understand the challenges of parents of children with CCN, the concept has been defined in different ways, none of which were grounded in how parents of children with CCN perceive this concept, except for the realm of type 1 diabetes. The variation among studies in how burnout is conceptualized leaves room for ambiguity and hampers the possibility of detecting and providing accurate care and support to parents who may experience burnout. Furthermore, to identify the root causes of burnout and prevent misunderstanding and confusion, appropriate understanding of the concept is essential. The context of parents of children with CCN differs from the context of parents of children without CCN [33], which may, apart from quantitative differences in care tasks, also require insight into qualitative differences related to their unique experiences. To address this gap, we selected a varied group of parents based on parental, child, and family characteristics and asked them to share their perspectives on what burnout means in their role as parents of children with CCN.

## 2 Methods

### 2.1 Study procedures

Parents were recruited through several parental organizations and schools for children with CCN and social media. Parents were invited to participate “in a study regarding the experiences on burnout or burnout related symptoms in parents of children with CCN”. Parents were eligible if they had a child with CCN, described as “...those who have or are at increased risk of chronic physical, developmental, behavioral, or emotional conditions and who also require health and related services of a type or amount beyond that required by children in general” (McPherson et al. [34], p. 138), and if they recognized or identified themselves with burnout-related symptoms based on their own perceptions and assumptions (irrespective of having or not having experienced a burnout). Hence, no definition of burnout or list of potential complaints or symptoms that could signify burnout was given because our aim was to obtain a comprehensive understanding of how parents themselves conceptualize burnout. Furthermore, to prevent interference with recovery from burnout and professional help, participants were excluded if they had received any psychiatric treatment related to burnout in the past six months, were on a waitlist for such treatment, or evidenced suicidal ideation.

We employed purposive sampling to select a varied group of parents, considering factors such as parental gender, ethnicity, child age, type of CCN, sibling status, and living arrangements. Once the data collection started, a snowball sampling method was applied to identify more potential participants. A more detailed description of the methods used in this study is provided in the Open Science Framework (OSF) protocol and can be accessed via: https://osf.io/xd8t5/?view_only=21ce436f383b40f0904f9910678b8578.

After including the first 15 parents, additional participants were included until the analysis of three additional interviews did not generate new content or insights, such as codes or nuances of the meaning of the codes [35]. Saturation was determined jointly by NP, KM, and AW, who participated in each step of the data analysis and were familiar with the data. Ethical approval was obtained from the Scientific and Ethical Review Board of the Faculty of Behavioral and Movement Sciences of the Vrije Universiteit Amsterdam (registration numbers: VCWE-2020-147 and VCWE-2021-078).

### 2.2 Semi-structured interviews and the PBI

An interview topic list was created by the research team, which included experts of experience and was pilot tested with a parent of a child with CCN. The topic list consisted of open-ended questions. Two partially overlapping topic lists were used, one for those participants who identified as having experienced burnout and one slightly briefer version for those who recognized burnout symptoms but did not identify themselves as having experienced burnout. The topic lists can be found in Appendix 1. Video interviews were conducted through Microsoft Teams or Zoom between May 2021 and June 2023. Each interview involved two interviewers with varying compositions consisting of undergraduate students, a researcher, experience-expert parents, or a healthcare professional working with parents of children with CCN. All the interviewers were trained by NP. One interviewer led the interview, while the other managed the timing and occasionally asked probing questions. Each participant was interviewed once, with a maximum duration of 60 min, and all interviews were audio recorded.

Each participant was asked to complete the Parental Burnout Inventory^1^ online [14] within two days prior to their scheduled interview. The PBI was employed to assess the variation in burnout scores, serving as an indicator for the variation in burnout-related symptoms and feelings among participants. The PBI has been validated in the Netherlands among parents of children without CCN [36]. It comprises 22 questions covering three dimensions: (1) emotional exhaustion (EE) (eight items, e.g., “I feel emotionally drained by my parental role”), (2) emotional exhaustion (ED) (eight items, e.g., “I do not really listen to what my children tell me”), and (3) personal accomplishment (PA) (six items, e.g., “I look after my children's problems very effectively”). Response categories range from 0 (never) to 6 (every day). A higher score indicates higher levels of parental burnout symptoms, except for PA, where the score is reversed, and a lower score signifies higher levels of parental burnout. The internal consistency in the present study was α = 0.95 for EE, α = 0.73 for ED, α = 0.73 for PA, and α = 0.90 for the whole PBI. The participant PBI scores can be found in Table 1.

**Table 1:** Demographic characteristics and PBI scores of participants (*N* = 38).

*Parent*	n (%)	*M* (*SD*)	min-max
Mothers	30 (79)		
Age at interview in years		44.34 (8.29)	30–74
Ethnicity western⁎	34 (90)		
Employed (paid)	31 (82)		
*Family*			
Married/ Cohabiting	33 (87)		
1 child with CCN	32 (84)		
≥ 2 children with CCN	6 (16)		
Child with CCN (partially) living at home with parent⁎⁎	31 (84)		
*Child*			
Age of child with CCN in years		12.66 (10.23)	1–47
Motor or sensory disability/ impairment	6 (14)		
Chronic or severe illness	8 (18)		
Intellectual and developmental disorders	21 (48)		
Comorbid conditions	9 (20)		
*PBI scores*			
Total score (possible range 0–128)		38.39 (19.41)	0–85
Emotional Exhaustion (0–46)		20.53 (13.29)	0–46
Emotional Distancing (0–46)		10.32 (6.37)	0–31
Personal Accomplishment (0–36)		7.55 (5.02)	0–20

⁎ Western encompasses participants descending from North America, Oceania, Indonesia, Japan or Europe (excluding Turkey) [40] Nonwestern parents included individuals from South America (n = 1), West Asia (*n* = 1), East Asia (n = 1), and North Africa (n = 1).”

⁎⁎ Based on *n* = 37.

The interviewers assured the participants that they could request taking a break or end the interview as necessary. If participants were distressed during or after the interview, interviewers were instructed to ask if they had someone to confide in and, if needed, suggest contacting their general practitioner for extra support. Informed consent was obtained from all participants.

### 2.3 Qualitative analysis

The semi-structured interviews were transcribed verbatim, pseudonymized and then analyzed using the six phases of inductive thematic analysis proposed by Braun and Clarke [37]. These six phases were conducted in different compositions of the project group (see reflexivity paragraph 2.4) to ensure different perspectives and prevent early convergence. First, NP and two graduate students read and familiarized themselves with the transcripts, making initial notes of possible codes and themes. Then, one transcript was open coded for pilot testing. The transcript was then discussed by NP, KM, AW, the graduate students and a healthcare professional from the project group to understand alternative coding and interpretations of the text fragments, to encourage thoroughness in the coding process and to establish general rules for open coding. Following this exercise, all interviews were coded by two independent coders using ATLAS.ti 23 for Mac [38]. Second, when approximately 70% of the transcripts were open coded, two transcripts were randomly drawn to create subthemes. The subthemes were collaboratively created by combining open codes into categories. After all the transcripts had been open coded, NP read through all the open coded transcripts, checked the initial open codes, and added or adjusted the open code when necessary and then categorized all the open codes into the agreed-upon subthemes. New subthemes were created when needed. Third, once all the transcripts had been coded according to subthemes, NP, KM, and AW used the list of subthemes to generate initial themes in Excel. Thereafter, and fourth, the initial themes were further refined, and potential connections between themes were discussed with the project group. The collaborative discussion took place in Miro [39]. Finally, respondents received a draft of the results and were encouraged to reflect on whether their perspectives and experiences were accurately represented in the draft.

### 2.4 Reflexivity

The project team consisted of eight people with expertise in psychology, special education, quantitative and qualitative research, intellectual and physical disability, rehabilitation, and nursing. Two of the project members were experts by experience (KM, MV), two were healthcare professionals working with families of children with CCN, and four were researchers with different levels of experience in conducting research (NP, AW, MK, CS). Five students (three graduate and two undergraduate students) with diverse educational and ethnic backgrounds participated in the data collection. NP and the students involved in data collection and analysis had limited prior experience with research in parents of children with CCN compared to other team members. Continuous reflective discussions occurred throughout the research phases.

## 3 Results

### 3.1 Participant characteristics

Seventy-one parents showed interest in participating in the study. One was ineligible, eight did not respond to repeated contact attempts, and 24 parents were not included because they would not increase variation or data saturation. Thirty-eight parents participated in the study. The diagnosis of the children with CCN varied from mild developmental challenges to severe, life-threatening conditions. The outcomes of the PBI showed variation in burnout scores across the domains measuring burnout symptoms among the parents (see Table 1).

### 3.2 Key themes

We identified three themes on how parents conceptualized burnout: (1) long-term and recurring; (2) from stress to exhaustion; and (3) survival mode. The themes are described in the following sections, along with illustrative quotes identified by the notation “P” for the parent and “#” for the corresponding number.

#### 3.2.1 Long-term and recurring

Burnout was perceived as a multifaceted concept. The boundary between being in a state of burnout and experiencing only burnout-related symptoms could not be articulated based on the data. How burnout presents itself varied from person to person. Burnout was, however, often seen as a long-term and recurring phenomenon and, as such, differed in terms of intensity and duration from mere stress or overexertion. Many parents described that being in and recovering from a burnout would take many years. Others questioned whether they ever truly recovered from burnout and expressed that they would experience better days and then worse days, making burnout-related feelings a recurring phenomenon:

*“I do feel that the stress symptoms probably never completely disappeared. Maybe for a period they did. But it just feels like once you've gone through that phase [referring to burnout], your nerves are just broken or something. Like it never fully heals, or at least you have some kind of scar that makes it reopen quickly. I don't know if I should describe it that way. Like an old wound reopening or something.”* [P18].

The recurring and long-term manifestation of burnout among parents of children with CCN was attributed to their unceasing responsibility for their child, leaving them with no choice but to endure feelings of depletion and being out of energy. One parent depicted this as follows:

*“It's like you're constantly driving on reserve fuel. That red light is always on. If I'm lucky, I can occasionally add just enough fuel to keep going with that red light on. But I never have a full tank. I just can't fill it up. I don't have the time for that. It's just not possible. It gets used up faster than you can replenish it.”* [P32].

#### 3.2.2 From stress to exhaustion

Several parents of children with CCN described three phases in the deterioration of state, leading to full burnout. Burnout commenced with (1) symptoms of stress that evolved into (2) overexertion and ultimately culminated to (3) exhaustion. A wide array of symptoms was mentioned, which were not exclusive to a particular phase but varied in intensity and per person. Seven subthemes were identified. An overview of exemplar symptoms per subtheme can be found in Table 2.

**Table 2:** Sub themes within the theme “From stress to exhaustion” and examples of associated symptoms.

*Sub theme*	Symptoms
Fatigue and sleep disturbances	Lack of energy, inability to relax, sleep-related problems, being fatigued and constantly tired after extensive sleeping
Impaired functioning	Struggling to handle and execute daily activities, unable to perform at preferred level, feeling of being at odds with oneself
Physical complaints	Heart palpitations, headaches, hyper ventilation, back- and neck pain, feeling swollen, weakened immune system and thereby being repeatedly ill
Cognitive complaints	Issues with concentration, difficulties thinking clearly and maintaining focus, issues processing information, inability to remember simple things
Difficulties in regulating emotions	Anxiety, panic, restlessness, tension, emotional suppression, being overwhelmed with emotions
Depressive feelings	Being melancholic, not being able to enjoy things, lack of interest in things
Exhaustion	Collapsing (mentally and physically)

*Fatigue and sleep disturbances* Many parents described burnout as being extremely tired, as one parent stated *“...And that I was intensely tired. Even when I had slept well, I woke up tired.”* [P33]. The tiredness was often related to extensive caregiving for their poorly sleeping child, who often experienced discomfort or pain. Caregiving tasks extended beyond typical parenting duties and persisted past the early years of the child's life. Additionally, worry for the child and uncertainties regarding the future for the child, the parent and the whole family also contributed to sleep disturbances.

*Impaired functioning* Some parents noted that the demands of caregiving made them struggle to handle and execute daily activities, such as maintaining their roles, which occurred when fulfilling their parental or professional obligations became progressively challenging, or when they found themselves unable to perform at their preferred level. Impaired functioning also resulted in the feeling of being at odds with oneself.

*Physical complaints* A wide array of physical complaints was mentioned. Some complaints were attributed to the status of their child, as one parent mentioned: *“The stress he experiences [referring to child with CCN] is immediately transferred to me. It's a physical stress that makes my heart rate increase. My blood pressure goes up too.”* [P11].

*Cognitive complaints* The constant demands of caregiving made some parents experience cognitive difficulties, which made some feel as if they lost track of their lives. These complaints often extended to daily tasks beyond caring for the child and involved, for example, excessive rumination or inability to remember simple things.

*Difficulties in regulating emotions* Parents pointed out feeling intense emotions, which were difficult to control. Some mentioned emotions that accompanied their chronic sorrow. Several parents highlighted feeling irritable (short fuse) toward others and at times toward the child. Some noted flashbacks of traumatic events from childbirth and from their own youth. Some parents explained that they would suppress emotions, while others explained being flooded with emotions, leading to bouts of crying. Difficulties regulating emotions also led some to crave unhealthy foods.

*Depressive feelings* Several parents mentioned feeling depressed and melancholic, linked to the ongoing demands of caregiving, uncertainties about the future for the child, and their unceasing responsibility. Parents also pointed out that they were not able to enjoy things they once did and generally felt a lack of interest in things. Furthermore, contemplating existential questions was not uncommon within this theme.

*Exhaustion* Parents mentioned feeling exhausted, which was often described as the final stage of persistent stress and overexertion, typically viewed as the point of collapsing. At this stage, parents felt that everything was too much and too difficult to handle, making it challenging to preserve. One parent described this overwhelming feeling: *“The feeling that I could not fight for my son. I didn't feel anymore connected with the world. I was just done.” [P4]* Parents also conveyed that it was at this point that they recognized their struggle and inability to effectively manage the situation.

#### 3.2.3 Survival mode

Several parents described being in a “survival mode”. Within the survival mode, we distinguished two subthemes: (1) fighting and (2) flighting. Parents described being in survival mode as a fluctuation between fighting and fleeing.

*Fighting* Parents described fighting as being in a constant state of heightened alertness. One parent illustrated this as being a “mama bear”. Furthermore, parents mentioned that they projected an image of having everything under control, often referring to being able to perform daily tasks such as working, managing the child's care and well-being, as well as their own. As one parent remarked:

“*… I had fallen behind in my household chores, but I really wanted to show the outside world that I could still manage everything, that I could juggle all the demands. I even put on a floral dress just to appear a bit cheerful. But, I, yeah, I wasn't myself anymore, I was really just surviving*.” [P10].

Some parents explained that they were in this state because they did not set boundaries, which was often linked to the feeling that they had a compelling duty to persevere, as there would be no one else who could take responsibility for caring for their child with CCN.

*Fleeing* Parents described fleeing as a survival mechanism in which they mentally and/or physically detached and distanced themselves from their situation. This distancing was characterized by feelings of numbness, a sense of not being oneself, and a disconnection from the world. Some parents also mentioned distancing themselves from people in their social network and even from their own children. This meant, for some, that they focused solely on the functional aspects of caring for their child while disconnecting emotionally from their children. As one parent described:

*“...to also not really be present with the children, actually, it was more like, well, you make sure there's food and drinks, and that the house is clean, and you help this one and that one, and everything is fine. But actually, the involvement is completely gone. Yes, even the enjoyment is also gone.”* [P19].

Some parents also expressed a desire to physically distance themselves from their circumstances or indulge in fantasies to end or escape their situation, underscoring the potentially isolating nature of being is this mode. Fantasies of ending the situation encompassed thoughts of resignation, suicidal ideation, or harboring destructive thoughts concerning their child. Fantasies of escaping the situation often involved seeking refuge in their work as a safe haven and a distraction from their harsh reality at home:

“*...but the main source of stress you get from, that's still there and that's home. Yeah. I don't think it's uncommon for parents in our category, so to speak, to say how wonderful it is to go to work for a while. Because it's so peaceful there. And that I said back then as well [referring to when experiencing a burnout], even though my work was quite intense and heavy. I thought it was wonderful to go to the office. Wonderful to get out of the house. And that sounds very harsh. But that's the reality, unfortunately.*” [P16].

When in the survival mode, some mentioned that they were unaware of the dire situation at the time, while others in their close network would occasionally notice.

## 4 Discussion and conclusion

### 4.1 Discussion

With semi-structured interviews and thematic analysis, this study investigated how parents themselves conceptualize burnout when their children have CCN. Three themes were found: “long-term and recurring”, “from stress to exhaustion”, and “survival mode”, which only partially overlap with existing dimensions in research on burnout outside the context of parenting a child with CCN.

Considering the first theme “long-term and recurring”, several parents explained that burnout commonly manifested as a persistent and repetitive phenomenon. This facet has not been incorporated into existing conceptualizations of burnout. The reason why this phenomenon occurs among parents of children with CCN appears to be the long-term dependence of children on consistent care and attention. The current sample included parents of children with various conditions, such as intellectual developmental disorders, which manifest in early childhood and typically require lifelong and often increasing levels of care. Even if parents may recover from burnout-related thoughts and feelings, the enduring sense of responsibility means that these parents may sooner or later re-enter the pathway toward burnout. This finding aligns with the conclusions drawn by Abdoli et al. [32], who similarly observed that relinquishing responsibilities is not considered an option for parents of children with CCN. To curb long-term, recurrent burnout, assessment and support may therefore take the parents' history of burnout-related thoughts and feelings into account. Another implication is that families may need more long-term support that is responsive to changes in care that are needed for children with CCN. Furthermore, considering this long-term and recurring nature, conceptualizations of burnout that emphasize a qualitative distinction in dichotomous categories (i.e., a diagnosis which can be present or not) may not fully capture the complexity of the context of parenting a child with CCN. This implies that further development of measures of burnout in the context of parenting a child with CCN might be needed.

The second theme, “from stress to exhaustion”, captures that burnout evolves across successive phases, commencing with individual variations in symptoms of stress, followed by overexertion and culminating in exhaustion. This facet aligns with the current literature that also views burnout as a consequence of persistent stress [12]. Furthermore, exhaustion has been identified as a dimension in most conceptualizations of burnout [21,22], and as a core dimension of parental burnout [14]. In the current literature, however, exhaustion is conceptualized around emotional responses, while parents of several children with CCN also included physical exhaustion in their conceptualization of burnout (e.g., headaches, back-pain, repeatedly being sick). Some parents mentioned attributing physical complaints to the wellbeing of their child. However, another potential reason for this could be that parents of children with physical disabilities or comorbidities, which were also present in our sample, engaged in physically heavy caregiving tasks (Vadivelan et al., 2020). Furthermore, prolonged exposure to stressors may result in psychosomatic complaints [41]. Cognitive difficulties and feelings of fatigue were also identified as symptoms, which corresponds with previous studies [16,18]. Other previously identified facets of burnout were also described by parents, such as depressed feelings and impaired functioning [24,42]. Underlying this wide array of symptoms is the unceasing long-term responsibility of parents of children with CCN for their child, causing a potential perpetual cycle of stress, overexertion, and exhaustion. To alleviate or mitigate burnout, it may help to first recognize that these states are part of a cycle. Second, work with families may involve the identification of risk and resilience factors and redressing their balance. Research on risk and protective factors may also help to differentiate between the causes and consequences of burnout and may help to delineate the burnout construct.

In the third and final theme, parents conceptualized burnout as being in a “survival mode”, trying to project an image to themselves and others of having the demands of caregiving for a child with CCN under control (fighting), while distancing physically and emotionally from others and themselves (fleeing). ‘Fighting’ and ‘fleeing’ partially overlap with extant conceptualizations of burnout. ‘Fighting‘could be seen as part of the dimension ‘personal accomplishment’, which concerns parental effectiveness [14,24]. However, parents in our study explained that they projected accomplishment to others but did not necessarily feel that way themselves. As a result, burnout may also be hidden from others. To our knowledge, this aspect has not been described in the scientific literature on burnout. ‘Fleeing’ resonates largely with the well-described dimension of emotional distancing from the child [14,24]. However, we found that distancing was not limited solely to emotional detachment from the child but extended to distancing oneself from others and oneself as a parent and physically withdrawing from the situation. Distancing might decrease possibilities for asking and accepting support, requiring the environment to continuously and persistently indicate that help is available. The overall description of the survival mode has been acknowledged in the grey literature on burnout within parenting [43,44] and aligns with the nonscientific construct of ‘burn-on’, which is considered to be the functional form of ‘burnout’ where one presents having complete control over the situation, while in the meantime experiencing an inner void, self-alienation, and feelings of meaninglessness [45]. However, the context in which parents of children with CCN experience burnout is different from that of parents in general due to the long-term dependence of children.’Burn-on’ or other constructs may be further explored as alternatives that might capture the experience of parents of children with CCN better than the construct of burnout.

This study does not come without limitations. First, the study involved only Dutch-speaking parents living in the Netherlands. The findings are therefore bound to their specific cultural context, social expectations of parenting, and attitudes toward children with CCN. Future research on burnout within the context of parenting a child with CCN should be conducted across cultural contexts, thus supporting interventions tailored to families and communities that are open to variations in meaning assigned to the concept. Second, only parents who identified with the term participated in the study. Throughout the recruitment process. We noticed that some parents refrained from participating because they did not want to be associated with the term. It is therefore also important to be open to potentially stigmatizing aspects of terms such as parental burnout. This raises the question of what burnout means for those parents who reject the term and raises the prospect of exploring a more suitable term to describe experiences similar to burnout. This could be an intriguing area for future research.

### 4.2 Innovations

This study is innovative because it is the first study to broaden the understanding of what burnout entails from the perspective of parents of children with CCN. This approach revealed new aspects of burnout that have not previously emerged in the literature on parental burnout, supporting the idea of a context-specific approach to burnout among parents of children with CCN. As themes were raised by parents themselves, discussing these themes with parents may provide a way to support them in reflecting on their own thoughts and feelings, which may arise from caring for a child with CCN. The results of this study are also innovative because our approach involved perspectives of parents of children with a variety of care needs. Indeed, attending to the individual subjective experiences of parents can shed new light on issues such as burnout, and this expectation was borne out in the current study. Furthermore, the approach of this study is innovative because it was part of a project that is coordinated, executed, and supported by parents of children with CCN, together with researchers and professionals with expertise on this topic. The productive interactions in this team led to a focus on understanding the meaning that parents themselves prescribe to burnout, a term utilized by parents, professionals and other experts within the field of parenting.

### 4.3 Conclusion

Parents of children with CCN conceptualized burnout as a long-term, recurring state, marked by a process from stress to exhaustion while also being in survival mode. Burnout was not only multifaceted but also manifested itself in parents in unique ways and to different degrees. The findings emphasized the need for a long-term perspective, both for understanding the origins and consequences of burnout and for prevention and intervention. Burnout in the context of parenting a child with CCN shows specific caregiving and parenting aspects, such as the long-term responsibility over the child, which cannot be relinquished, and the ‘hidden’ nature, which refers to the image parents may want to project of everything being well and under control. Therefore, it may be difficult to detect burnout. With this article, we hope to raise societal awareness regarding this phenomenon, as well as awareness among parents and professionals, to encourage open discussion and thereby address burnout.

## Funding

This work was supported by the Netherlands Organization for 10.13039/100005622Health Research and Development [grant number 641001103] and Stichting Wetenschappelijk Onderzoek ‘s Heeren Loo.

## Declaration of Generative AI and AI assisted technologies in the writing process

During the preparation of this work the author(s) used ChatGPT in order to improve readability and language and for translation purposes, as well as Curie to correct grammar mistakes and typos. After using these tools/services, the author(s) reviewed and edited the content as needed and take(s) full responsibility for the content of the publication.

## CRediT authorship contribution statement

**Nathalie J.S. Patty:** Conceptualization, Data curation, Formal analysis, Project administration, Supervision, Validation, Writing – original draft, Writing – review & editing, Funding acquisition, Methodology, Investigation. **Karen M. van Meeteren:** Conceptualization, Formal analysis, Funding acquisition, Investigation, Methodology, Project administration, Supervision, Validation, Writing – original draft, Writing – review & editing. **Minke Verdonk:** Conceptualization, Formal analysis, Funding acquisition, Investigation, Methodology, Project administration, Writing – review & editing. **Marjolijn Ketelaar:** Conceptualization, Formal analysis, Funding acquisition, Methodology, Supervision, Validation, Writing – review & editing. **Carlo Schuengel:** Conceptualization, Formal analysis, Funding acquisition, Methodology, Supervision, Validation, Writing – review & editing. **Agnes M. Willemen:** Conceptualization, Formal analysis, Funding acquisition, Methodology, Supervision, Validation, Writing – original draft, Writing – review & editing.

## Declaration of competing interest

Financial support was obtained from Stichting Wetenschappelijk Onderzoek ‘s Heeren Loo for the co-author Carlo Schuengel. The funding sources had no role in the study design, data collection, analysis and interpretation of the data, and in writing the report.

## Appendix A Supplementary data

Supplementary material Interview topic lists Image 1

## Footnotes

1 MBI - Human Services Survey - MBI-HSS: Copyright ©1981 Christina Maslach & Susan E. Jackson. All rights reserved in all media. Published by Mind Garden, Inc., http://www.mindgarden.com

## References

[1] Baker K., Claridge A.M. (2022). “I have a Ph.D. in my daughter”: Mother and Child Experiences of Living with Childhood Chronic Illness. J Child Fam Stud.

[2] Woodgate R.L., Edwards M., Ripat J.D., Borton B., Rempel G. (2015). Intense parenting: a qualitative study detailing the experiences of parenting children with complex care needs. BMC Pediatr.

[3] Kirk S., Glendinning C. (2002). Supporting ‘expert’ parents – professional support and families caring for a child with complex health care needs in the community. Int J Nurs Stud.

[4] Page B.F., Hinton L., Harrop E., Vincent C. (2020). The challenges of caring for children who require complex medical care at home: ‘The go between for everyone is the parent and as the parent that’s an awful lot of responsibility,’. Health Expect.

[5] Cousino M.K., Hazen R.A. (2013). Parenting stress among caregivers of children with chronic illness: a systematic review. J Pediatr Psychol.

[6] Hatzmann J., Heymans H.S.A., Ferrer-i-Carbonell A., van Praag B.M.S., Grootenhuis M.A. (2008). Hidden consequences of success in pediatrics: parental health-related quality of life—results from the care project. Pediatrics.

[7] Senses Dinc G., Cop E., Tos T., Sari E., Senel S. (2019). Mothers of 0–3-year-old children with down syndrome: effects on quality of life. Pediatr Int.

[8] Kütük M.Ö., Tufan A.E., Kılıçaslan F., Güler G., Çelik F., Altıntaş E. (2021). High depression symptoms and burnout levels among parents of children with autism spectrum disorders: a multi-center, cross-sectional, case–control study. J Autism Dev Disord.

[9] Scherer N., Verhey I., Kuper H. (2019). Depression and anxiety in parents of children with intellectual and developmental disabilities: a systematic review and meta-analysis. PloS One.

[10] Mroskova S., Reľovská M., Schlosserová A. (2020). Burnout in parents of sick children and its risk factors: a literature review. Cent Eur J Nurs Midwifery.

[11] Patty N.J.S., van Meeteren K.M., Willemen A.M., Mol M., Verdonk M., Ketelaar M. (2024;33;1378-1392). Understanding burnout among parents of children with CCN: a scoping review informed by a stakeholder consultation. J Child Fam Stud.

[12] Mikolajczak M., Roskam I. (2018). A theoretical and clinical framework for parental burnout: the balance between risks and resources (BR2). Front Psychol.

[13] Mikolajczak M., Brianda M.E., Avalosse H., Roskam I. (2018). Consequences of parental burnout: its specific effect on child neglect and violence. Child Abus Negl.

[14] Roskam I., Raes M.E., Mikolajczak M. (2017). Exhausted parents: Development and preliminary validation of the parental burnout inventory. Front Psychol.

[15] Sánchez-Rodríguez R., Perier S., Callahan S., Séjourné N. (2019). Revue de la littérature relative au burnout parental [review of the change in the literature on parental burnout]. Can Psychol.

[16] Lindahl Norberg A. (2007). Burnout in mothers and fathers of children surviving brain tumour. J Clin Psychol Med Settings.

[17] Lindahl Norberg A., Mellgren K., Winiarski J., Forinder U. (2014). Relationship between problems related to child late effects and parent burnout after pediatric hematopoietic stem cell transplantation. Pediatr Transplant.

[18] Lindström C., Åman J., Norberg A.L. (2011). Parental burnout in relation to sociodemographic, psychosocial and personality factors as well as disease duration and glycaemic control in children with type 1 diabetes mellitus. Acta Paediatr.

[19] Lindström C., Åman J., Norberg A. (2010). Increased prevalence of burnout symptoms in parents of chronically ill children. Acta Paediatr.

[20] Zeeshan M., Shaikh S., Ahmer Z. (2022). How frequent is burnout among informal caregivers of disabled children? Findings from a cross-sectional study in Karachi, Pakistan. Child Care Health Dev.

[21] Maslach C., Jackson S.E. (1981). The measurement of experienced burnout. J Organ Behav.

[22] Melamed S., Ugarten U., Shirom A., Kahana L., Lerman Y., Froom P. (1999). Chronic burnout, somatic, arousal and elevated salivary cortisol levels. J Psychosom Res.

[23] Mikolajczak M., Aunola K., Sorkkila M., Roskam I. (2023). 15 years of parental burnout research: systematic review and agenda. Curr Dir Psychol Sci.

[24] Roskam I., Brianda M.E., Mikolajczak M. (2018). A step forward in the conceptualization and measurement of parental burnout: the parental burnout assessment (PBA). Front Psychol.

[25] Alrahili N. (2023). Burnout and anxiety among parents of children with neurodevelopmental disorders: a cross-sectional study in Saudi Arabia. Middle East Curr Psychiatry.

[26] Gérain P., Zech E. (2018). Does informal caregiving lead to parental burnout? Comparing parents having (or not) children with mental and physical issues. Front Psychol.

[27] Liu S., Zhang L., Yi J., Liu S., Li D., Wu D. (2023). The relationship between parenting stress and parental burnout among Chinese parents of children with ASD: a moderated mediation model. J Autism Dev Disord.

[28] Roskam I., Mikolajczak M. (2023). Parental burnout in the context of special needs, adoption, and single parenthood. Children.

[29] Kwiatkowski P., Sekułowicz M. (2017). Examining the relationship of individual resources and burnout in mothers of children with disabilities. Int J Spec Educ.

[30] Naeem F., Afir S., Asghar A., Mahmood Z. (2018). Psychosocial stressors, burnout and mental health problems in caregivers of children with cerebral palsy. J of Postgrad Med Inst.

[31] Sadziak A., Wiliński W., Wieczorek M. (2019). Parental burnout as a health determinant in mothers raising disabled children. Balt J Health Phys Act.

[32] Abdoli S., Vora A., Smither B., Roach A.D., Vora A.C. (2020). I don’t have the choice to burnout; experiences of parents of children with type 1 diabetes. Appl Nurs Res.

[33] Tadema A.C., Vlaskamp C. (2010). The time and effort in taking care for children with profound intellectual and multiple disabilities: a study on care load and support. Br J Learn Disabil.

[34] McPherson M., Arango P., Fox H., Lauver C., McManus M., Newacheck P.W. (1998). A new definition of children with special health care needs. Pediatrics.

[35] Hennink M.M., Kaiser B.N., Marconi V.C. (2017). Code saturation versus meaning saturation: how many interviews are enough?. Qual Health Res.

[36] Van Bakel H.J.A., Van Engen M.L., Peters P. (2018). Validity of the parental burnout inventory among dutch employees. Front Psychol.

[37] Braun V., Clarke V. (2021).

[38] Scientific Software Development GmbH (2023).

[39] Maplesoft, Miro. https://www.miro.com 2023 (accessed 6 November 2023).

[40] CBS (2023). Person with a western migration background. https://www.cbs.nl/en-gb/our-services/methods/definitions/person-with-a-western-migration-background.

[41] Yaribeygi H., Panahi Y., Sahraei H., Johnston T.P., Sahebkar A. (2017). The impact of stress on body function: a review. EXCLI J.

[42] Ahola K., Hakanen J., Perhoniemi R., Mutanen P. (2014). Relationship between burnout and depressive symptoms: a study using the person-centred approach. Burn Res.

[43] COPE Centre of Perinatal Excellence, Parental burnout. https://www.cope.org.au/new-parents/emotional-health-new-parents/parental-burnout/ 2023 (accessed 8 November 2023).

[44] Feller B.S. (2021). Don't be a bad mom: Burnout in American mothers of young children. https://www.diva-portal.org/smash/get/diva2:1587869/FULLTEXT02.pdf.

[45] Lannoey M. (2022).

