# Supplementary material for: Conceptualizing burnout from the perspective of parents of children with complex care needs
Source: PEC Innov. 2024 Jul 17;5:100325. doi: 10.1016/j.pecinn.2024.100325 (PMC11332200; doi:10.1016/j.pecinn.2024.100325)
Supplement: Supplementary file 1 — Supplementary material Interview topic lists [file mmc1.docx]

**Topic list for parents who identify with having experienced a burnout**

1. Can you briefly tell us about your child and their disability?

**Experience regarding burnout**

1. When did you experience burnout and how often?
2. How would you describe your burnout? What does it look like?
3. How did your burnout begin? What were the warning signs?

***Circumstances causing burnout**

1. According to you, what circumstances/factors played a role in experiencing burnout? What caused your burnout?
2. Of the circumstances you just described, which one played the most significant role in your case?

***Coping with burnout/overcoming it**

1. According to you, what helped you overcome burnout? How did you manage to come out of it?
2. What did you learn from the time you had burnout?
3. How do you ensure that you won't experience burnout again?
4. What advice would you give to other parents to keep on going?
5. We have come to the end of our questionnaire. Is there anything you would like to add that we haven't asked about or any questions you would like to ask us?

**Topic list for parents who recognize burnout related symptoms but have not experienced a burnout**

1. Can you briefly tell us about your child and their disability?

**Experience regarding burnout**

1. How would you describe burnout? What does it look like?
2. Do/did you experience burnout-related symptoms related to caring for your child?

***Circumstances causing burnout**

1. According to you, what circumstances play a role in getting burnout?
2. Of the circumstances you just described, which one do you think plays the most significant role?

***Coping with burnout-related symptoms**

1. How do you deal with burnout-related symptoms?
2. How do you ensure that burnout-related symptoms do not resurface?
3. According to you, what circumstances/factors are relevant in preventing burnout?
4. Of the circumstances/factors you just described, which one do you believe plays the most significant role in preventing burnout?
5. What advice would you give to other parents to be able to keep on going?
6. We have come to the end of our questionnaire. Is there anything you would like to add that we haven't asked about or any questions you would like to ask us?

* NOTE. This study is part of larger qualitative research on burnout among parents of children with CCN investigating also contextual circumstances attributing to burnout and the preventive and restorative factors mitigating or alleviating burnout, according to parents of children with CCN. The shaded grey text belonged to second part of this larger study.
